# Virus transmission from urinals

**DOI:** 10.1063/5.0021450

**Published:** 2020-08-01

**Authors:** Ji-Xiang Wang, Yun-Yun Li, Xiang-Dong Liu, Xiang Cao

**Affiliations:** 1 College of Electrical, Energy and Power Engineering, Yangzhou University, Yangzhou 225009, People’s Republic of China; 2 Key Laboratory of Energy Thermal Conversion and Control of Ministry of Education, School of Energy and Environment, Southeast University, Nanjing 210096, People’s Republic of China

## Abstract

A virus-laden particle movement from urinal flushing is simulated. Similar to the toilet-induced flushing, results indicate that the trajectory of the particles triggered by the urinal flushing manifests an external spread type. Even more alarmingly, the particle can reach 0.84 m (man’s thigh) in 5.5 s when compared with the diffusion performance of the toilet-induced one (around 0.93 m in 35 s). A more violent climbing tendency is discovered in this Letter. Wearing masks should be made mandatory in public washrooms, and anti-diffusion improvements of facilities in public washrooms are urgently needed, especially in the current “SARS-CoV-2” crisis.

Late last year, a huge outbreak of the COVID-19 occurred across the world.^1^ At this point, millions of people have been confirmed to be infected by the novel corona virus called “SARS-CoV-2,” causing huge economic loss and panic worldwide. It has been confirmed that one of the main transmission approaches of the SARS-CoV-2 is the respiratory droplet transmission.^2,3^ In addition, many recent publications are devoted to blocking of such transmission.^4,5^ In addition, fecal-oral transmission has been identified as a probable transmission route for the “SARS-CoV-2” as the virus can be detected from feces of confirmed cases,^6^ which arouses public attention for better toilet usage. Li *et al.*^7^ integrated Volume of Fluids (VOF) and Discrete Phase Model (DPM) to investigate the toilet flushing process. The results show that the strong turbulent caused by the flushing flow would lift the virus originally in the toilet bowl to a high position. It implies that the toilet-based “SARS-CoV-2” could cause cross-infection among people and teaching the public to use the toilet properly is mandatory. What is worse, two of the COVID-19 reemerging confirmed cases in Beijing have been reported to be infected from a public toilet,^8^ which practically proves the danger from the public restroom.

Recently, researchers extracted the virus particle of “SARS-CoV-2” from urine of a confirmed case of COVID-19, which means urine-based transmission could be a previously ignored transmission route.^9^ It means that besides the toilet, the male-oriented urinal, which is a common facility in the male public restroom, could become another dangerous item. However, there are few relevant studies focusing on the health problems brought from the urinal. “Can a male-oriented urinal promote the virus transmission?” is the question needed to be answered with the assistance of computational fluid dynamics (CFD) in this Letter as urinal flushing, where turbulence can be observed from daily life experiences, occurs frequently as well.

Figure 1 presents the outline and size information of the focused urinal and its adjacent air region. The black line in Fig. 1 shows the outline of the urinal, and the red lines mark the boundary of the adjacent air region. The wall represents the interface of the solid urinal surface and its internal air. The gray and purple surfaces in Fig. 1 are the flushing inlet and outlet 1, respectively, where the flushing water is poured in and drained out of the urinal correspondingly. Outlet 2 is composed of the faces composed of the red line. The overall structured mesh condition of the object is displayed in Fig. 2, where the local enlarged images of the encrypted meshes of the wall, the inlet, and outlet 2 are also given. The total mesh number is 901466. Mesh sensitivity analysis was conducted, which shows that a mesh number of 901466 can obtain both computational accuracy and relative economy.

**FIG. 1.**
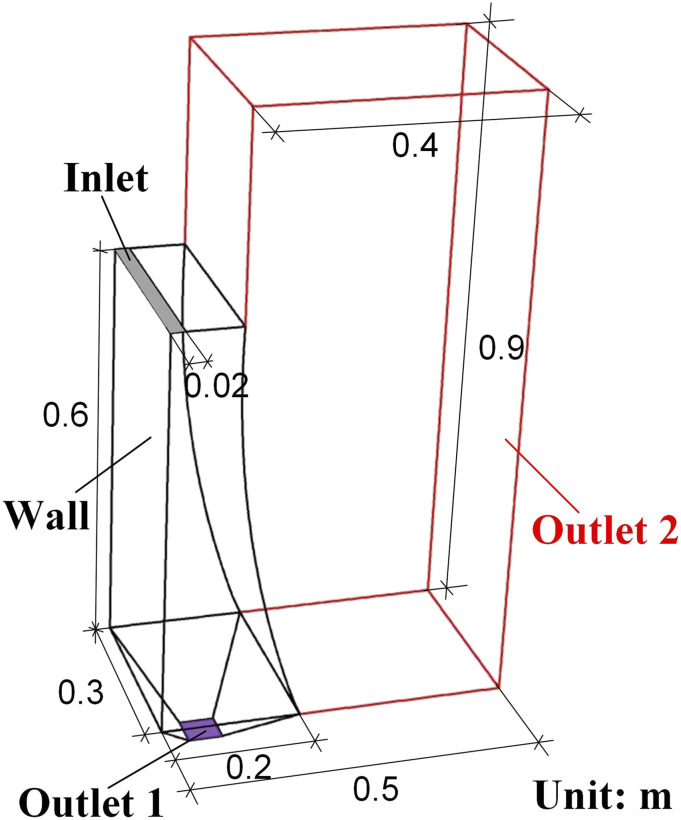
Structure and size information of the focused urinal and adjacent air region.

**FIG. 2.**
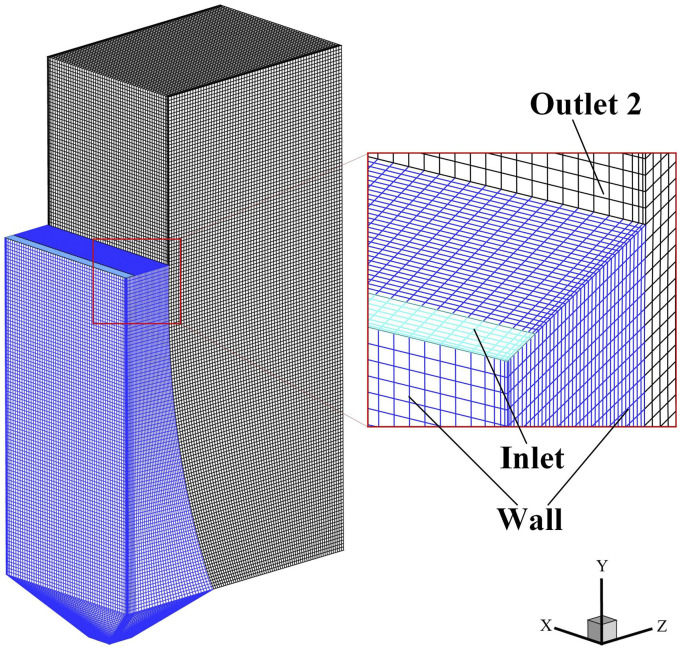
Mesh condition of the focused object.

Similar to the flushing of the toilet, the flushing process of the urinal involves significant interactions between the gas and liquid interfaces. Therefore, this Letter adopts the VOF model^10^ to track and characterize the two-phase interface^11^ in which the realizable *k*–*ε* turbulence model is accepted. In addition, the flow pattern of virus aerosols under the urinal flushing is reflected by the DPM model, a Lagrangian scheme, which has successfully simulated the human cough-induced flow^12^ and sprayed droplet flow.^13,14^ Please refer to the work by Li^7^ for the detailed math formulations.

During simulation, several assumptions are adopted: (1) there are no heat and mass (evaporation) interactions between the particles and the air and liquid phases; (2) generation of the aerosol particles during the flushing is ignored; (3) physical properties and size of the aerosol particles are constant; and (4) the temperature remains to be 20 °C. The initial condition of the aerosol particle distribution in the urinal is shown in Fig. 3. Information on the CFD simulation relations and boundary conditions is the subject of our previous study.^7^

**FIG. 3.**
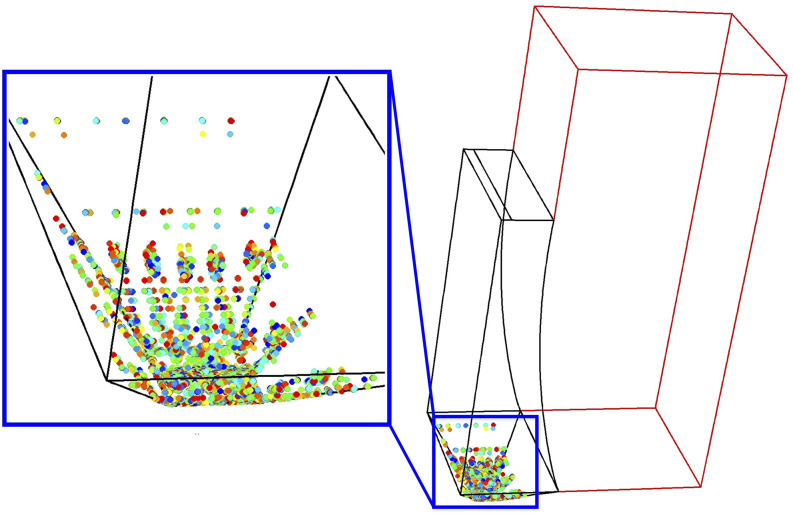
Initial condition of the aerosol particle distribution of the focused urinal.

Figure 4(a) shows that there are strong Y-component velocity fluctuations for the focused point as violent liquid-flow interactions occur when the water touches the bottom. In addition, as shown in Fig. 4(b), a large amount of vorticity is formed with the maximum vorticity magnitude up to 2007.74^−1^, manifesting a strong turbulence. The vorticity also moves out of the urinal due to the effect of the vorticity diffusion effect. As shown in Fig. 4(a), the maximum Y-component velocity can reach +4.62 m/s for point I (0, −0.64, 0.04) at 0.9 s, which appears to be the maximum vorticity magnitude at that time. The results here suggest that an alarming upward flow with strong turbulence can be generated.

**FIG. 4.**
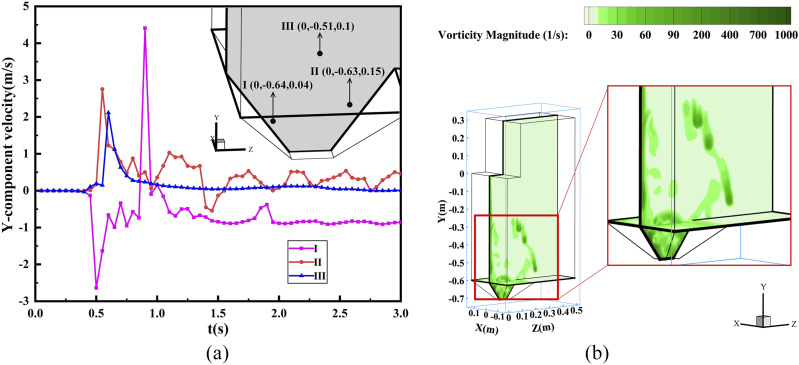
Results of urinal flushing: (a) transient Y-component velocity and (b) vorticity magnitude distribution at 0.9 s.

Y–Z velocity, which is presented in Fig. 5, can quantitatively describe the outward spread trend of the airflow from the urinal. The gravity-induced downward flushing water results in a negative Y–Z velocity near the inside wall of the vertical urinal. The effects of inertial force and centrifugal force make a positive Y–Z velocity in the lower domain inside the urinal. Comparing the images of Figs. 5(a)–5(d) along the time line, the area with positive Y–Z velocity expands continuously. The maximum Y–Z velocity value of 3.61 m/s appears at 0.5 s. It is reasonable to predict that the urinal flushing would cause a large spread of potential aerosol particles residing in the urinal originally.

**FIG. 5.**
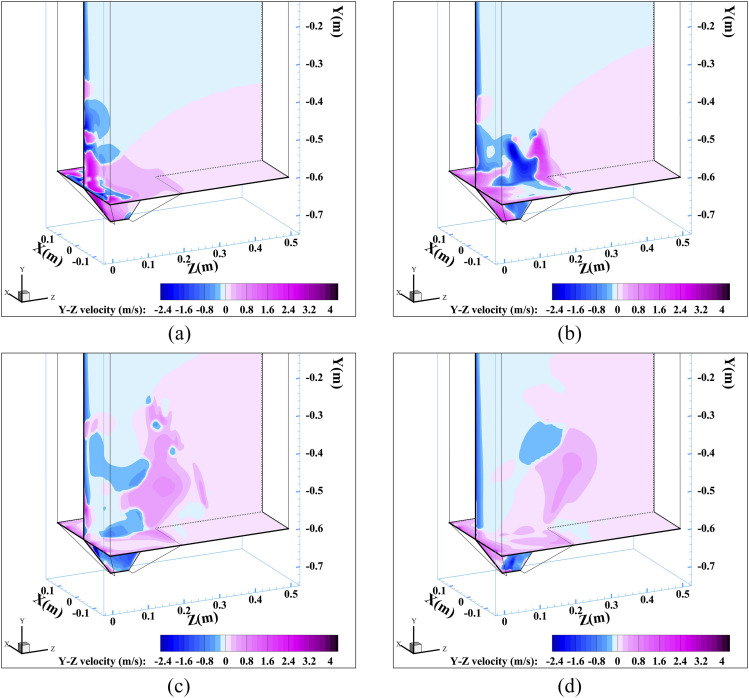
Transient Y–Z velocity at different times: (a) 0.5 s, (b) 0.6 s, (c) 0.9 s, and (d) 1.45 s.

Transient virus particle movement during and after urinal flushing is provided in Fig. 6 (multimedia view), where the flushing was initiated at 0 s and finished at 2.6 s. The particle distribution at 2.6 s is illustrated in Fig. 7, where numerous particles have been spread out of the interior–exterior interface (IEI) of the urinal. At the time of 3.8 s, that is, in the post-flushing period (1.2 s has passed since the end of the flushing), the diffused particle has touched the edge of the computational domain, meaning the farthest particle has traveled nearly 0.04 m in the Z-position. At the time of 5.5 s, the highest vertical position can be 0.84 m, assuming that the distance between the urinal bottom and the ground is 0.05 m. The outward velocity (Z-component velocity) and climbing velocity (Y-component velocity) can reach 0.186 m/s and 0.235 m/s, respectively, at the end of the simulation (5.5 s) when more than 57% of the total number of particles have passed through the IEI. It can be predicted that in public restrooms, especially those in densely populated areas, urinals are used more frequently and particles will travel faster and fly farther, which poses a great challenge to the public health.

**FIG. 6.**
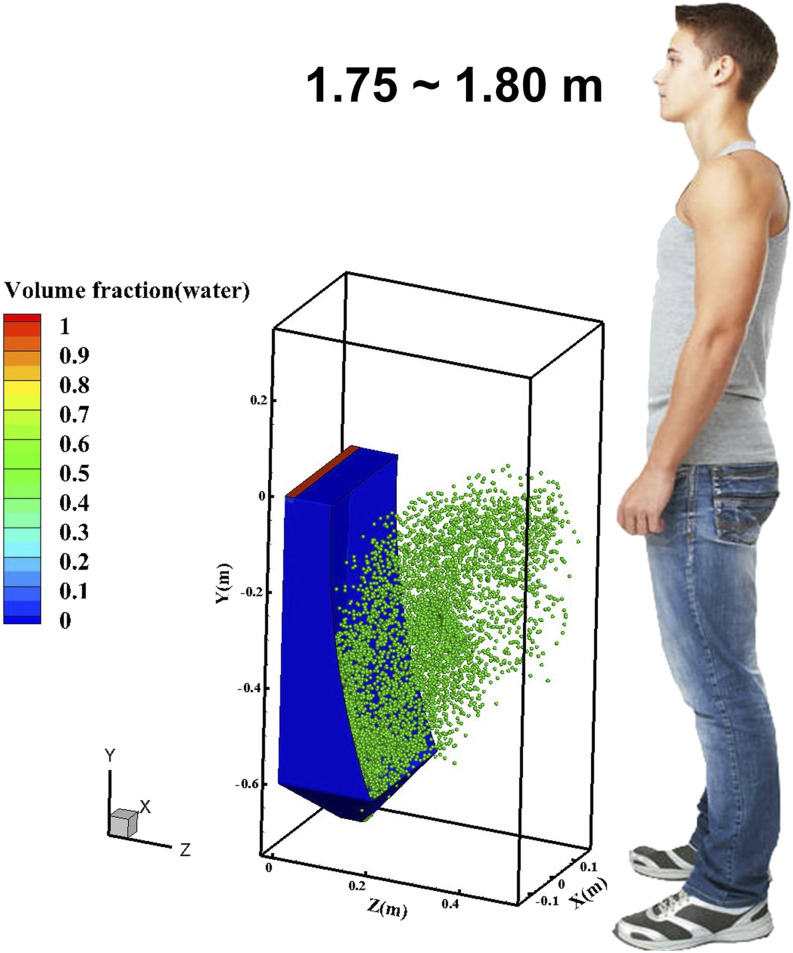
Dynamic virus movement during and after a 2.6 s urinal flushing with a total duration of 5.5 s. Multimedia view: https://doi.org/10.1063/5.0021450.110.1063/5.0021450.1

**FIG. 7.**
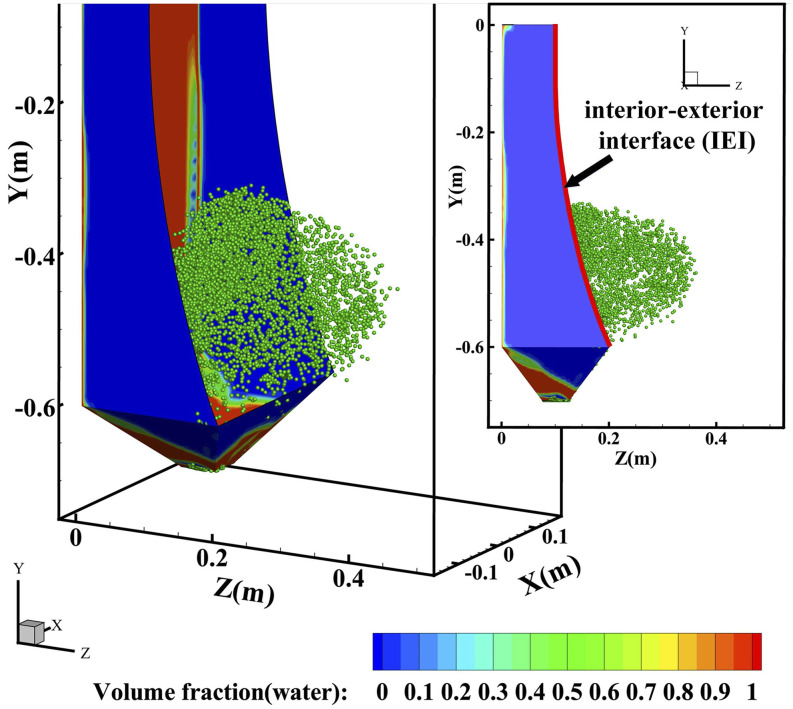
Discrete particle distribution of the urinal flushing at 2.6 s.

In summary, enlightened and invited by Editor Alan Jeffrey Giacomin, a virus-laden particle movement with urinal flushing is simulated in this Letter. Similar to toilet flushing, alarming results are discovered: (1) more than 57% of the particles have traveled away from the urinal; (2) constant diffusion tendency is uncovered due to relatively considerable diffusion velocity; (3) only in 5.5 s, the highest position of 0.84 m is reached, where the climbing speed is significantly higher than in the toilet-induced diffusion. According to this Letter and our previous contribution,^7^ wearing masks when in public washrooms should be mandatorily implemented.

## DATA AVAILABILITY

The data that support the findings of this study are available from the corresponding author upon reasonable request.
